# The handling of SARS-CoV-2 associated deaths - infectivity of the body

**DOI:** 10.1007/s12024-021-00379-9

**Published:** 2021-06-02

**Authors:** Ann Sophie Schröder, Carolin Edler, Benjamin Ondruschka, Klaus Püschel, Julia Schädler, Axel Heinemann, Fabian Heinrich, Marc Lütgehetmann, Susanne Pfefferle, Martin Aepfelbacher, Antonia Fitzek, Jan-Peter Sperhake

**Affiliations:** 1 Institute of Legal Medicine, University Medical Center Hamburg-Eppendorf, Butenfeld 34, 22529 Hamburg, Germany; 2 Institute of Medical Microbiology, Virology and Hygiene, University Medical Center Hamburg-Eppendorf, Martinistraße 52, 20251 Hamburg, Germany

**Keywords:** Coronavirus, SARS-CoV-2, COVID-19, Autopsy, Infectivity, External Examination

## Abstract

The body of a deceased with Severe Acute Respiratory Syndrome Coronavirus 2 (SARS-CoV-2) infection is considered infectious. In this study, we present the results of infectivity testing of the body and testing of mortuary staff for SARS-CoV-2. We performed real-time quantitative polymerase chain reaction (RT-qPCR) for SARS-CoV-2 on 33 decedents with ante mortem confirmed SARS-CoV-2 infection. Swabs of the body surface from five different body regions and from the body bag or coffin were examined. A subset of the swabs was brought into cell culture. In addition, screening of 25 Institute of Legal Medicine (ILM) personnel for ongoing or past SARS-CoV-2 infection was performed at two different time points during the pandemic. Swabs from all locations of the body surface and the body environment were negative in cases of negative post mortem nasopharyngeal testing (n=9). When the post mortem nasopharyngeal swab tested positive (n=24), between 0 and 5 of the body surface swabs were also positive, primarily the perioral region. In six of the cases, the body bag also yielded a positive result. The longest postmortem interval with positive SARS-CoV-2 RT-qPCR at the body surface was nine days. In no case viable SARS-CoV-2 was found on the skin of the bodies or the body bags. One employee (autopsy technician) had possible occupational infection with SARS-CoV-2; all other employees were tested negative for SARS-CoV-2 RNA or antibody twice. Our data indicate that with adequate management of general safety precautions, transmission of SARS-CoV-2 through autopsies and handling of bodies is unlikely.

## Introduction

The body of a deceased person with ante mortem confirmed infection with severe acute respiratory syndrome coronavirus 2 (SARS-CoV-2) is considered contagious. It has been shown that PCR-based postmortem detection of SARS-CoV-2 RNA is possible and that viable (and thus potentially infection-causing) virus can also be found in cadavers [1–6]. The predominant routes of transmission of SARS-CoV-2 via droplet infection and aerosols are unlikely to be important in non-invasive handling of a deceased person (e.g. external necropsy, coffining). Consideration should be given to smear and contact infections from the body fluids that may be present in the mouth, on the skin, and surrounding of the body.

However, autopsies and ablutions of SARS-CoV-2 decedents may produce infectious droplets or aerosols. To date, we are not aware of any safely confirmed occupational SARS-CoV-2 infections of autopsy teams during autopsies of SARS-CoV-2 positive decedents [7–10]. Since the beginning of the pandemic, the potential infectivity of the body has led to widespread adoption of a restrictive approach to deaths associated with coronavirus disease 2019 (COVID-19), avoiding any contact with the body. In the interim, there have been several recommendations. Dijkhuizen et al. provide an overview of various recommendations in the management of SARS-CoV-2 associated deaths [11]. However, there are no legally binding rules for the management of SARS-CoV-2 associated deaths.

Despite initial recommendations released in the spring of 2020 to avoid autopsies due to the risk of infection, numerous autopsies have been performed at the Institute of Legal Medicine in Hamburg (ILM) of the University Medical Center Hamburg-Eppendorf (UKE) since the first COVID-19 deaths occurred in Hamburg. Many confirmed SARS-CoV-2 infected decedents, as well as suspected cases, have been investigated [12–14]. Handling of known COVID-19 deaths was performed with extended personal protective equipment, including category 2 or 3 filtering face pieces (FFP2, FFP3), disposable gloves, and protective gowns. For autopsies, additional eye protection, head covering, and protective footwear were worn by all personnel. Autopsies were performed in a separate autopsy room equipped with a table exhaust device, low-pressure ventilation, and an air exchange circulation system with a minimum of 10 air changes per hour. The cranial cavity was opened using an oscillating saw with suction, or a hand saw. In deceased who tested positive for SARS-CoV-2 incidentally, often no masks, or only a simple mouthguard (as opposed to FFP2- or FFP3-Masks) was worn during the external examination.

In this study, we present the results of our COVID-19 cadaveric infection investigations and a staff test for SARS-CoV-2. The aim was to investigate the relevance and effectiveness of infection control measures in the management of SARS-CoV-2-associated deaths in Hamburg, Germany.

## Methods

**Fig. 1:**
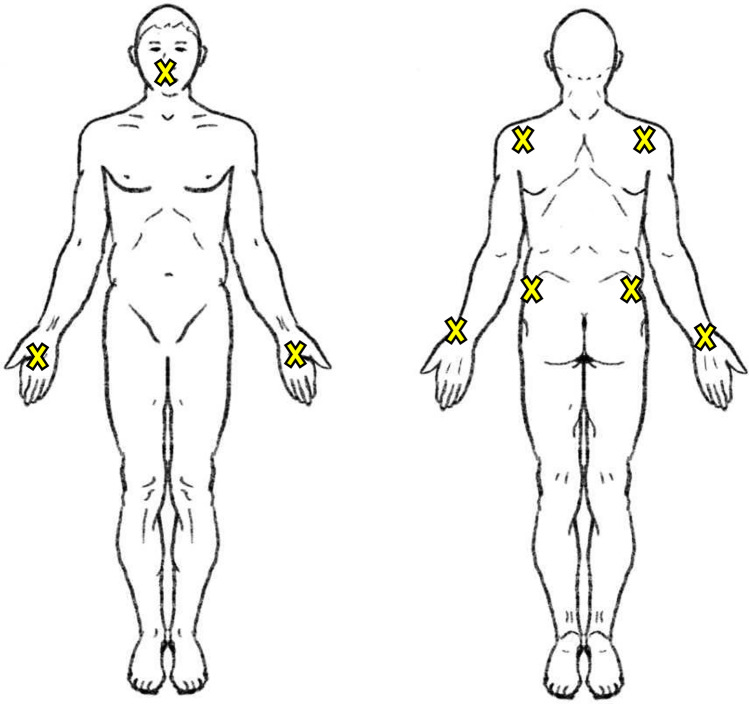
Location of the swaps taken from the skin of the body

Between June 2, 2020, and November 15, 2020, 33 decedents who were tested positive for SARS-CoV-2 ante mortem and who were clinically considered to have died of COVID-19 were examined post mortem at the ILM. A nasopharyngeal swab and swabs from the following regions were collected prior to autopsy or other invasive procedures or cleaning: 1 - body bag or coffin, 2 - perioral, 3 - hands (dorsal and palmar), 4 - wrists, and 5 - shoulder and hip regions (combined) (Fig. 1). If the deceased was wrapped in two body bags, the outer body bag was examined. If there was no body bag but a coffin, the latter was examined externally. Gloves were changed after each swabbing. To avoid further contamination, all contact with the body and surfaces was avoided. For the external surfaces (region 1 to 5), the swab (COPAN eSwab™, Brescia, Italy) was moistened in the liquid transport medium of the swab tube before collection. Swabs were collected with circular movements and gentle pressure for 15 s. The swabs were examined as part of routine diagnostic procedures at the Institute of Medical Microbiology, Virology and Hygiene (IMV) of the UKE. Detection and quantification of viral ribonucleic acid (RNA) was performed by quantitative real-time polymerase chain reaction (RT-qPCR) as previously described [15–18].

Between October 27, 2020 and November 15, 2020, the swabs of regions 1 to 5 were collected twice from 15 of the deceased patients. RT-qPCR for SARS-CoV-2 RNA was performed in one swab and the second swab was stored at -80°C. When RT-qPCR results were positive, cell culture experiments of the second swab were set up in a biosafety level 3 (BSL-3) laboratory in 30 specimens out of 11 cases.

For virus viability testing, 500 µl of each swab medium (modified Amies medium) was used for virus adsorption onto a well of a 24-well plate containing ~ 80% confluent Vero cells (ATCC CCL-81) for 1 h. Cells were then washed once with phosphate-buffered saline (PBS) and fresh DMEM (Dulbecco's Modified Eagle's Medium containing 3% fetal calf serum, 1% penicillin–streptomycin, 1% L-glutamine [200 mM], 1% sodium pyruvate, and 1% non-essential amino acids [all from Gibco/Thermo Fisher Scientific, Waltham, MA, USA]) was added. After incubation at 37 °C for 72 h, virus growth was analyzed as previously described [19].

The following data were collected on our cohort: Age, place of death (outpatient vs. inpatient), postmortem interval (PMI) between death and swab collection. SARS-CoV-2 RNA loads of the swabs were categorized as follows: Negative (-), low positive (+ : < 1000 copies/ml), positive (+ + : 1000–100,000 copies/ml), and high positive (+ + + > 100,000 copies/ml). Data were analyzed descriptively and statistical analysis was performed using GraphPad Prism software version 9.0.0 (GraphPad Software, CA, U.S.A.).

After 3½ months of daily work with SARS-CoV-2 positive decedents at ILM and several crematoria, including external examinations and 163 conventional autopsies, 25 ILM personnel were tested for active or past SARS-CoV-2 infection. RT-qPCR was performed from a throat swab in universal transport medium as previously described [15]. SARS-CoV-2 antibodies (IgG/IgM/IgA) directed against the viral nucleocapsid were tested using the Cobas e411 system (Roche Diagnostics, Manheim, Germany) according to the manufacturer's recommendations. To confirm positive serologic reactions, we used an orthogonal testing strategy as recommended by the Centers for Disease Control and Prevention (USA) for regions with SARS-CoV-2 prevalence below 5%. Therefore, a second assay was performed targeting the viral spike protein (Anti-SARS-CoV-2 (S1/S2), Liaison XL, DiaSorin, Saluggia, Italy). A second round was performed six months later. These analyses were performed at the IMV of the UKE. The following data were collected from the employees: Sex, age, position, and symptoms of general illness in the last seven days before the tests. The data were analyzed descriptively.

## Results

The results of body and body environment testing for SARS-CoV-2 are shown in Table 1. The mean age of the cohort of 33 decedents was 79 years (range 55–99 years). Seventeen patients died during hospitalization, 16 in the outpatient setting. On average, swabs were collected 3.5 days postmortem (range 0–17 days). Overall, 24 deceased (72.7%) tested RT-qPCR positive for SARS-CoV-2 in the postmortem nasopharyngeal swab (highlighted in orange in Table 1), indicating SARS-CoV-2 infection at the time of death, and nine tested negative (highlighted in blue in Table 1). Consistently, in all cases with RT-qPCR-negative nasopharyngeal swabs, all other swabs from the body surface and cadaveric environment were also negative. In cases with positive postmortem nasopharyngeal swab, most frequently perioral swabs were confirmed positive (21/24; 87.5%). In six of these cases, the body bag tested positive (at least > 1000 copies/mL). SARS-CoV-2 RNA loads in swabs from the immediate cadaveric environment were found to correlate significantly with nasopharyngeal viral loads, as shown by simple linear regression (region 2 - perioral: p < 0.0001, region 3 - hands: p = 0.0005, region 4 - wrist: p = 0.0005, region 5- shoulder/hip: p = 0.002). However, the viral RNA loads detected on the body bags did not significantly correlate with the nasopharyngeal SARS-CoV-2 RNA loads of each body (site 1: p = 0.10). The longest PMI with a positive test result from the body surface was 9 days in two cases, while this PMI was examined in three individuals. One of the deceased also tested positive at regions 2 to 5, and the other case was weakly positive at the perioral region.

Viable virus could not be detected in any of the 30 specimens examined in cell culture with viral RNA loads of < 1000 up to > 100,000 (Table 1).

The results of screening employees for active or past SARS-CoV-2 infection are shown in Table 2. After 3½ months of the ongoing pandemic, no SARS-CoV-2 infection or SARS-CoV-2 antibodies were detected in any of the ILM employees. Six months later, RT-qPCR testing for SARS-CoV-2 infection was still negative in all 25 employees tested, but IgM antibodies to SARS-CoV-2 were detected in one employee (an autopsy technician). However, it remains unclear whether transmission occurred in a private or occupational context.

## Discussion

SARS-CoV-2 infections are usually transmitted by droplet infection and aerosols. However, transmission of the virus via surfaces is possible in principle. The stability of coronaviruses in the environment depends on temperature, humidity, surface properties, virus quantity, and the virus strain. Specifically for SARS-CoV-2, Chin et al. showed high stability at temperatures of 4 °C, with more rapid inactivation observed at increasing temperatures (inactivation in the medium at 37 °C after two days, after five minutes at 70 °C); high stability was also shown at pH values between 3 and 10 and high humidity [20]. When handling bodies, the relevant surfaces are wood (coffin), plastic (body bag), textiles, and skin. In addition, there may be a particular risk of transmission through infectious body fluids. Under laboratory conditions, viable viruses have been detected in three studies on wood between 24 h and 7 days after contamination, on plastic between 3 and 7 days, and on textiles or clothing between 24 h and 4 days [20–22]. The study by Liu et al. consistently showed longer survival times of the virus on all surfaces compared to the other two studies. One reason for this could be the higher amount of virus used for in vitro contamination. In practice, shorter viral survival times are more likely due to UV radiation, temperature fluctuations, as well as varying humidity and a lower amount of virus. In a study by Harbourt et al. the stability of SARS-CoV-2 was tested on the skin of pigs, and of clothing placed on the pigs [23]. The virus was found to be stable on skin at 4 °C for the duration of the experiment of 14 days and on clothing for at least 96 h. This is similar to the temperature of refrigerated bodies, indicating a possible risk of infection emanating from the human body. At higher temperatures, inactivation occurred more rapidly - on skin after 96 h at 22 °C and 8 h at 37 °C [23]. Although conditions may be different in vivo, viral stability on skin underscores the need for continuous hand hygiene to minimize the possibility of viral transmission in daily routine.

In our study, in all cases with a negative postmortem nasopharyngeal swab, all other swabs from the body and body bag were also negative. In deceased cases with a positive postmortem nasopharyngeal swab, between 0–5 swabs from the body surface and cadaveric environment tested positive in RT-qPCR, most commonly in the perioral area. Nevertheless, we did not find viable virus on the skin of deceased COVID-19 patients or on surfaces, including body bags. Moreover, viable virus was not detected in any of the samples from bodies that had a short PMI (1 day) either.

Consistent with the distribution of RT-qPCR-positive test results in the nasopharyngeal and perioral areas, possible contamination via the nasopharyngeal-perioral route is one explanation for the SARS-CoV-2 detection on the other surfaces. The absence of viable virus in cell culture suggests that no infectivity emanated from the body surface, although SARS-CoV-2 RNA was detected by RT-qPCR. Nevertheless, infection via contaminated skin or other surfaces cannot be completely excluded.

There were limitations to this study. Firstly, samples were only collected from five body regions. However, these regions are the most exposed regions when handling a deceased person in daily practice and all other body regions are usually touched less frequently while examining, moving, or transporting a deceased. Secondly, the sample size was rather small and the cell culture experiments were only performed on a subset of 11 cases or 30 samples, respectively.

We believe that autopsies in COVID-19-related deaths are an indispensable tool for obtaining scientific data that can contribute critically to a deeper understanding of the disease [10, 24]. Nevertheless, there was considerable reluctance to perform autopsies, particularly at the beginning of the pandemic, which continues to some extent [25, 26]. This was partly because the risk of infection posed by bodies was difficult to assess. Concerns exist not only among physicians who perform autopsies, but also technical personnel and physicians who only perform external post mortems. In contrast to these concerns, however, there are also reports indicating that autopsy personnel are not actually exposed to any particular risk when protective measures are applied [7, 9].

Concerns about the handling of the potentially infectious body also relate to burial practices. Open casket burials are not permitted in some cases. Ritual washing and embalming of the deceased, which are common in various cultures and religions, are not performed. Baj et al. provide recommendations on precautions around burial [27]. ILM staff follow the guidelines of the Robert Koch Institute, Germany's central scientific institution in the field of biomedicine, which recommend FFP2 masks, eye and face protection, body protection including clean, long-sleeved, liquid-tight or impervious protective clothing, double-layered gloves, and appropriate work shoes [28]. However, in our daily routine, the recommended protective measures (e.g. FFP2) were not always fully applied while handling bodies that were incidentally known to be infected only after arrival at the ILM.

All bodies transported to the ILM were consecutively screened for SARS-CoV-2 upon arrival. 'Normal' means of protection in the morgue are body protection and gloves. This way of handling a body applies to the usual daily work of morticians and crematory workers. In our institute, since the beginning of the pandemic, there has been a suspicion that the risk of infection of COVID-19 deceased may have been overestimated. If protective measures are complied with, the risk of transmission should be minimal at best. Deceased persons with potentially aerogenously transmissible infectious diseases (e.g. tuberculosis) are autopsied daily by pathologists worldwide, taking safety precautions into account. Nevertheless, infections may occur in the autopsy room due to aerosol-forming measures. Ultimately, however, apart from the autopsy itself, the handling of a deceased person's body, e.g. during transport, careful washing, or burial, is likely to be relatively harmless ("a body does not cough").

In international comparison the ILM in Hamburg has autopsied a relatively large number of COVID-19 cases [12]. The ILM staff all tested negative for SARS-CoV-2 after 3½ months, even after exposure to numerous COVID-19 decedents. After 9½ months, one autopsy assistant tested positive for IgM antibody. The mode of transmission remains unclear. However, this occurred at a time when the disease was generally very prevalent in Germany.

In our cohort, we found no evidence of an increased risk of transmission of the infection to funeral personnel and health care professionals who have to handle SARS-CoV-2-infected deceased persons. The greatest risk likely comes from unprotected contact with relatives and the unavailability of infection control measures. However, further research is needed, particularly in the often overlooked funeral industry, to analyze, for example, the prevalence of SARS-CoV-2 antibodies among workers in this field and adherence to the use of protective measures in daily practice. It must be acknowledged that the department's own risk assessment was a rather optimistic estimate for which no evidence was available at the onset of the pandemic. In particular, little was known about the potential infectivity of surfaces and instruments. However, the present results underline the correctness of this assessment.

## Conclusion

Our data shows, that with reasonable management of general safety precautions, SARS-CoV-2 transmission risk from handling bodies, including external examinations and autopsies, is very low.

## Key Points

1. No viable virus was found on the skin of deceased COVID-19 patients nor surfaces, including body bags.
2. If usual safety precautions (personal protective equipment and appropriate autopsy room) are respected, there is no concern about performing full autopsies.
3. Open-casket farewells may also be permitted, although unprotected touching of the body should be avoided.
4. The infection risk from transporting an infected body in a body bag is low.
5. Washing and embalming procedures can be performed by trained personnel if safety precautions analogous to autopsies are taken.

**Table 1:**
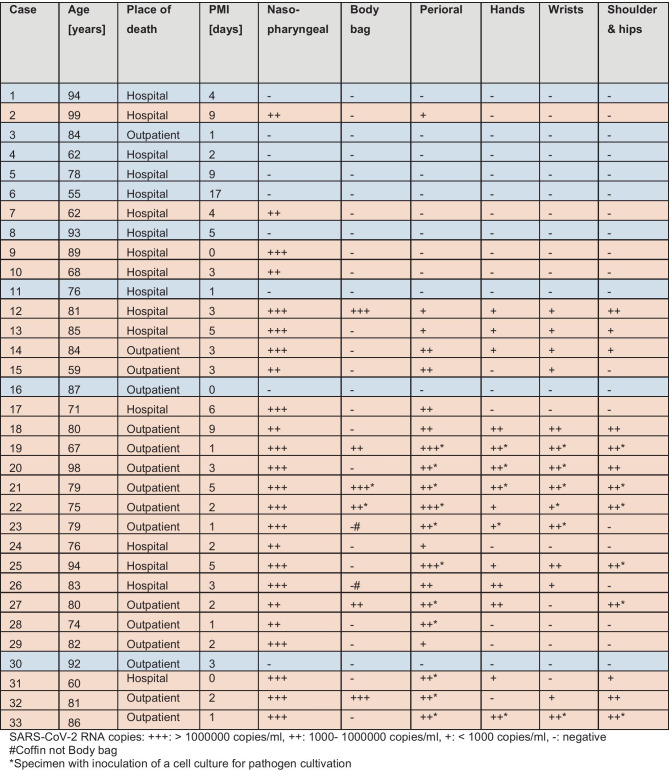
SARS-CoV-2 presence and copy numbers on the body and the body environment

**Table 2:**
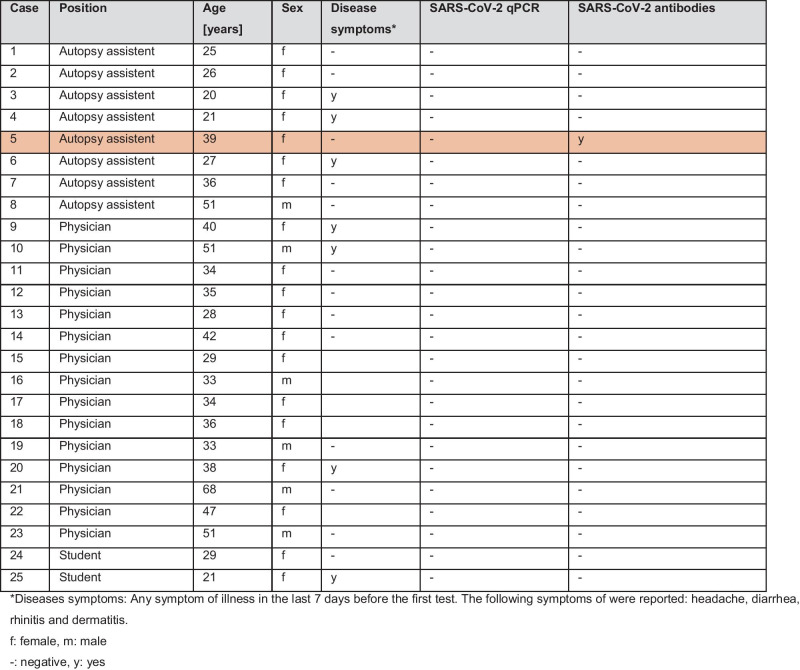
Testing of the employees for SARS-CoV-2
